# A Protein Interaction Information-based Generative Model for Enhancing Gene Clustering

**DOI:** 10.1038/s41598-020-57437-5

**Published:** 2020-01-20

**Authors:** Pratik Dutta, Sriparna Saha, Sanket Pai, Aviral Kumar

**Affiliations:** 1 Department of Computer Science and Engineering, Indian Institute of Technology Patna, Bihta, 801103 India; 2 Department of Chemical Science and Technology, Indian Institute of Technology Patna, Bihta, 801103 India

**Keywords:** Computational models, Machine learning, Microarrays

## Abstract

In the field of computational bioinformatics, identifying a set of genes which are responsible for a particular cellular mechanism, is very much essential for tasks such as medical diagnosis or disease gene identification. Accurately grouping (clustering) the genes is one of the important tasks in understanding the functionalities of the disease genes. In this regard, ensemble clustering becomes a promising approach to combine different clustering solutions to generate almost accurate gene partitioning. Recently, researchers have used generative model as a smart ensemble method to produce the right consensus solution. In the current paper, we develop a protein-protein interaction-based generative model that can efficiently perform a gene clustering. Utilizing protein interaction information as the generative model’s latent variable enables enhance the generative model’s efficiency in inferring final probabilistic labels. The proposed generative model utilizes different weak supervision sources rather utilizing any ground truth information. For weak supervision sources, we use a multi-objective optimization based clustering technique together with the world’s largest gene ontology based knowledge-base named Gene Ontology Consortium(GOC). These weakly supervised labels are supplied to a generative model that eventually assigns all genes to probabilistic labels. The comparative study with respect to silhouette score, Biological Homogeneity Index (BHI) and Biological Stability Index (BSI) proves that the proposed generative model outperforms than other state-of-the-art techniques.

## Introduction

One of the fundamental issues in the field of functional genomics is understanding the genes’ biological functionalities. Recent years have seen a rapid increase in studies into high-throughput techniques, particularly in the profiles of gene expression^1^. Analyzing gene expression values contributes to the exploration of certain biologically important genes and a stronger understanding of gene functions. Genes with analogous variations of expression have similar functionalities^2,3^. For the analysis of such data, clustering^4^ is a very popular unsupervised pattern classification method^5^. Clustering is an exploratory data analysis technique in which objects in the same cluster demonstrate greater resemblance than those which are in different clusters^6,7^. In the field of bioinformatics, gene clustering has a huge application in understanding molecular studies of the gene, disease gene classification task^8,9^ and also the design of new drugs^10^. This kind of analysis was first employed by Spang *et al*.^11^ and Golub *et al*.^12^. Since then, clustering methods have drawn a great deal of attention of Bioinformaticians^13^. Researchers have been proposing novel gene clustering methods by taking account of different intrinsic properties of the data^14,15^. In this regard, the necessity for developing an intelligent gene clustering system by utilizing the data pattern and functionalities is becoming crucial.

Now a days, using biological knowledge extracted from existing databases for gene clustering is the point of interest of the researchers. Gene Ontology^16^ is one such external resource that helps in improving gene clustering^17^. Also, it has been observed that the protein interaction information of the genes leverages the performance of a wide variety of biomedical tasks such as informative gene selection^18^, identification of the functional modules^19^, disease gene classification^8^, etc. Recently, protein interaction information has also shown promising results for improving gene clustering performance^20–22^.

With the advent of computational biology, there is an explosion of biomedical data. However, most of the data is unlabeled and noisy. Though, collection of a huge amount of unlabeled data is relatively easy, the validity of the results we obtain upon dealing with this data is highly questionable. Hence, relying on the unlabeled data may not be the right course of action in every situation. On the contrary, labeled data is more reliable than the unlabeled data. The main difficulties in acquiring labelled data are that the method is expensive and needs a great extent of human effort and knowledge. The collection of such labelled information is tremendously costly and we need experts (*subject matter experts* (SME)) in the field to develop this labelled information. While some large enterprises (https://www.wired.com/2016/11/googles-search-engine-can-now-answer-questions-human-help/, https://time.com/4631730/andrew-ng-artificial-intelligence-2017/) can bear this price^23^, it is not simple for most developers to bear the price.

There is a notable trend in using generative models^24^ to investigate data from *weak supervisory sources* to solve this bottleneck. These weak supervision sources which synthesize the labels by exploiting external knowledge bases^25^, heuristic laws^26^, noisy crowd labels^27^, or even other classifiers^28^, often have limited accuracy and coverage. As the labels are conflicting and noisy, these labels are not regarded to be gold standards. We must infer the dependence and correlation between them in order to solve this conflict. In this respect, the generative model plays an important role in inferring the probabilistic labels without having access to the ground truth. The user-specified structure of the generative model directly impacts the precision of the inferred labels^29–31^. Recently, the researchers of Stanford university proposed a new paradigm of generative model named Snorkel^28,32^. Due to the inherent property of Snorkel, it is widely used in various real life problems like surveillance with electronic health records^33^, clinical text classification^34^, web content and event classification^35^. Also, Snorkel is used for improving gene clusteing^36^ and medical image training^37^.

Motivated by the above stated facts, we utilized the generative model of Snorkel for developing a novel gene clustering technique. In this work, the final probabilistic labels of the genes are inferred by using protein interaction information, weak supervision sources and Snorkel. Recently, researchers have used generative model of Snorkel without modifying the internal architecture for improving the gene clustering^36^. In this study, the novel contribution is to integrate protein interaction information as a new parameter of the generative model of Snorkel. As per our knowledge, this type of integration of biological knowledge (protein interaction information) with generative model is a new and unique approach. Here, for generating weak supervised sources, we have utilized a multi-objective optimization (MOO) based clustering technique^20^ and Gene Ontology^38^. Recently, clustering methods based on multi-objective optimization^8,39^ have been discovered to be efficient in solving various real-life issues in clustering. The solutions of MOO-based clustering ${\Pi }^{(P)}=\left\{{\pi }_{1}^{(P)},{\pi }_{2}^{(P)},\ldots ,{\pi }_{M}^{(P)}\right\}$, present on the Pareto front are non-dominated to each other, i.e., $\left\{({\pi }_{i}^{(P)},{\pi }_{j}^{(P)})| {\pi }_{i}^{(P)}\ \prec \ {\pi }_{j}^{(P)}\ \wedge \ {\pi }_{j}^{(P)}\ \prec \ {\pi }_{i}^{(P)}\right\}$ where ≺ represents the dominance relation. Recently, the authors of^36^ utilize the non-dominated solutions as the weak supervised sources of the generative model. In the proposed approach, we prudently integrate protein interaction information with the generative model so that it can label the gene expression data efficiently. The protein interaction information acts as a parameter for the generative model that helps in improving the accuracy of the generative model. The final clustering solution is then evaluated by three cluster validity indices namely biological homogeneity index (BHI)^40^, biological stability index (BSI)^40^ and Silhouette index^41^. Experimental results indicate that the technique we propose achieves better outcomes than the state-of- the-art techniques. In short, the suggested strategy is a novel way of improving gene clustering from weak supervision sources, by utilizing the protein interaction information and a generative model. For the ease of understanding of the readers, the list of mathematical logic symbols that are used throughout the article is shown in Table 1.

**Table 1:** Glossary of variables and symbols used in the paper.

Logic Symbols	Values
Π^(*P*)^	Set of solutions at Pareto front
${\pi }_{M}^{(P)}$	*M*^*th*^ solution(partitioning) at Pareto front
${\mathbb{M}}$	Proposed model
*G*	Gene expression profile
*N*	Number of genes in the gene expression profile
*F*	Number of samples(features) in each gene
*g*_*i*_	*i*^*th*^ gene of the gene expression profile
$\widehat{G}$	Prepossessed gene expression profile
$\widehat{N}$	Number of prepossessed gene
*S*	Non-dominated solutions of the proposed multi-objective optimization based clustering
*D*	Number of non-dominated solutions
${\mathbb{MO}}$	Proposed multi-objective optimization based clustering technique
*L*_*i*_	Label of *i*^*th*^ non-dominated solution
*λ*	Weak label function
*p*_*θ*_	Proposed generative model
${\mathbb{G}}$	Factor graph
Λ	Label matrix of size $\widehat{N}\times (D\ +\ 3)$
*Y*	Vector of final probabilistic labels
*ϕ*	Factors of the factor graph
*θ*	Parameters of the factor graph
*P*	Pareto front
*α*_*ij*_	Confidence score of the interaction between the proteins *g*_*i*_ and *g*_*j*_
*K*	Number of cluster centers in a solution
*C*_*i*_	*i*^*th*^ cluster of any solution or partitioning
*f*	Objective function

The current paper is unique in the following ways:

- A protein interaction based generative model is used for improving the gene clustering. The model utilizes different weak supervision sources and infers a probabilistic clustering solution.
- In this study, for weak supervision sources we have used MOO-based solutions along with the three Gene Ontology-based solutions.

The remaining part of the article is structured as follows. In the subsequent section, first, we provide the comprehensive description of the experimental evaluation along with a brief analysis of the performance for the proposed generative model. The next section provides a brief overview of the weak supervision sources and the proposed generative model. Finally we conclude the article by stating the uniqueness and future scope of the work.

## Results

In this section, we analyze the performance of the proposed generative model when it is applied on the gene expression profile. In this section, firstly, we briefly describe the details of the datasets. Then we provide a comparative performance analysis of different algorithms with our proposed generative model. Finally, a comprehensive discussion is presented. In the discussion section, we have analyzed the performance of the developed model in an incremental way, i.e., new components are added one by one and the enhancements in performance are reported.

### Experiment results

In this section, we comprehensively evaluated the performance of proposed protein interaction based generative model on three real-life NCBI datasets. We have compared the performance of the proposed generative model with different state-of-the-art techniques. For the performance measures, we have calculated two bio-oriented cluster validity indices, namely, biological homogeneity index (BHI) and biological similarity index (BSI) along with a traditional cluster validity index named Silhouette index^41^. For comparing the performance of the proposed method with different existing works, we have considered traditional clustering techniques, one multi-objective optimization based clustering technique, a multi-objective based differential evolution (MODE)^42^ approach, and a cluster ensemble technique. For traditional clustering techniques, we have utilized two popular clustering techniques, namely K-means^43^ and a density-based clustering technique named DBSCAN^44^. For the multi-objective optimization based clustering technique, we utilized an existing MOO-based clustering algorithm^20^ where three objective functions are simultaneously optimized. The three objective functions are Fuzzy Partition Coefficient (FPC), PBM index and DB index. In this MOO-based clustering, we reported the best non-dominated solution for comparison purpose. We have also utilized a pairwise similarity based ensemble technique^45^ as a state-of-the-art comparing method. In MODE^42^, which is a multi-objective based differential evolution algorithm, two objective functions are simultaneously optimized.

Along with these state-of-the-art methods, we prudently integrate different parts with the generative model so that the cumulative performance of the architecture follows an incremental way. Simultaneously, we have reported the performance of the proposed architecture in each integration step. Firstly, we have integrated the MOO-based solutions using the generative model (${{\mathbb{M}}}_{1}$: **(MO + GM)**) of Snorkel. Here only the partitioning solutions produced by MOO based technique are considered as the weak supervised solutions. In the next step, we integrated the protein protein interaction information with the generative model. In this integrated model (${{\mathbb{M}}}_{2}$: **(MO + PPI + GM)**), we consider protein protein interaction information as a parameter *θ*^*ppi*^ that specifies the strength of the accuracy factor, *ϕ*^*Acc*^, in the generative model, *p*_*θ*_. Lastly, apart from MOO based solutions, three GO-based solutions are also utilized as the weak supervised solutions in the final integrated architecture (${{\mathbb{M}}}_{3}$: **(MO + PPI + GM + GO)**). As in the GO-based solutions, all the genes are not labelled; we did not exploit only GO-based solutions as the weak supervision sources.

The comparative analyses of the performance of the proposed generative model with different state-of-the-art methods are shown in Tables 2, 3 and 4. These tables illustrate the performance comparison in terms of BHI (Table 2), BSI (Table 3) and Silhouette score (Table 4). From these tables, it is evident that we modelled the whole architecture in a way so that addition of different modules follows an incremental way in terms of performance. In general, the final integrated generative model (${{\mathbb{M}}}_{3}$) obtained higher BHI and BSI values compared to other existing models. For example, in BCLL dataset, the BHI value of ${{\mathbb{M}}}_{3}$ is 0.361 which is 50.42%, 9.06% and 4.64% improvements over MOO-based ensemble technique, ${{\mathbb{M}}}_{1}$ model and ${{\mathbb{M}}}_{2}$ model, respectively. For ILD dataset, the final integrated generative model(${{\mathbb{M}}}_{3}$) attains a BHI score of 0.475 which outperforms MOO-based ensemble technique, ${{\mathbb{M}}}_{1}$ model and ${{\mathbb{M}}}_{2}$ model by 11.24%, 4.86% and 3.26%, respectively. For prostrate dataset, ${{\mathbb{M}}}_{3}$ model attains a BHI score of 0.451 which is 10%, 1.3% and 0.6% performance improvements over MOO-based ensemble, ${{\mathbb{M}}}_{1}$ and ${{\mathbb{M}}}_{2}$, respectively. Also, ${{\mathbb{M}}}_{3}$ model achieves the BSI scores of 0.994, 0.941 and 0.945 for BCLL, ILD and prostrate datasets, respectively.

**Table 2:** Comparative study with respect to biological homogeneity index(BHI); MO: MOO-based solutions, GM: Generative model, PPI: Protein interaction information, GO: Gene Ontology based solutions.

	B-CLL	ILD	Prostrate
K-means	0.163	0.395	0.379
DBSCAN	0.193	0.417	0.396
MODE	0.236	0.421	0.406
Best MOO-based solution	0.236	0.428	0.410
Ensemble Technique (MOO-based solution)	0.240	0.427	0.410
Ensemble Technique (MO + GM)	0.331	0.453	0.445
Ensemble Technique (MO + PPI + GM)	0.345	0.460	0.448
Ensemble Technique (MO + PPI + GO + GM)	**0.361**	**0.475**	**0.451**

**Table 3:** Comparative study with respect to biological stability index(BSI); MO: MOO-based solutions, GM: Generative model, PPI: Protein interaction information, GO: Gene Ontology based solutions.

	B-CLL	ILD	Prostrate
K-means	0.934	0.860	0.884
DBSCAN	0.986	0.839	0.879
MODE	0.987	0.905	0.892
Best MOO-based solution	0.989	0.908	0.902
Ensemble Technique (MOO-based solution)	0.989	0.926	0.935
Ensemble Technique (MO + GM)	0.989	0.936	0.941
Ensemble Technique (MO + PPI + GM)	0.992	0.938	0.944
Ensemble Technique (MO + PPI + GO + GM)	**0.994**	**0.941**	**0.945**

**Table 4:** Comparative study with respect to Silhouette index; MO: MOO-based solutions, GM: Generative model, PPI: Protein interaction information, GO: Gene Ontology based solutions.

	B-CLL	ILD	Prostrate
K-means	0.879	0.479	0.055
DBSCAN	0.404	0.336	0.057
MODE	0.845	0.517	0.062
Best MOO-based solution	0.901	0.510	0.065
Ensemble Technique (MOO-based solution)	0.901	0.516	0.070
Ensemble Technique (MO + GM)	0.934	0.534	0.073
Ensemble Technique (MO + PPI + GM)	0.928	0.567	0.073
Ensemble Technique (MO + PPI + GO + GM)	**0.941**	**0.569**	**0.076**

In conclusion, the analysis as mentioned above shows that the proposed integrated generative model obtains better performance in grouping the genes in terms of biological relevance. Also, to validate the effectiveness of the result, we did a biological analysis and a statistical test. The detailed description and results of these validations are reported in the Table 5, respectively.

**Table 5:** *p*-values of the proposed technique generated by Welch’s t-test for the biological homogeneity index(BHI) of different methods.

Datasets	K-means	DBSCAN	MODE	Best MOO solution	MOO-based Ensemble	Ensemble (MO + GM)	Ensemble (MO + PPI + GM)
**B-CLL chronic lymphocytic leukemia**	5.63E-053	1.85E-049	2.06E-44	2.05E-044	1.32E-044	3.98E-022	4.19E-013
**ILD Interstitial lung disease**	8.54E-036	6.87E-032	1.35E-29	3.45E-028	8.36E-028	8.08E-016	9.62E-014
**Prostrate**	3.43E-034	4.99E-033	5.55E-26	7.04E-025	5.01E-023	2.16E-03	2.24E-03

## Discussion

In recent years, the generative model has been extensively used in many fields, and their applications in the bioinformatics domain shows a promising direction. However, this powerful method was never utilized for gene clustering. In computational biology, grouping the same biologically expressed genes improves diagnosis, prognosis, and treatment of a particular disease. Also, it has been found that the use of integrated information extracted from different related biological datasets improves the specific biological task. In this regard, we have utilized protein interaction and Gene Ontology-based information for improving gene clustering. In this study, we logically integrated different biological information in different steps of the generative model so that a noticeable increment in performance can be observed in each level of integration.

Generally, a generative model generates a solution by considering the correlations and dependencies of the inputs. The correlation is inferred by stochastic gradient descent (SGD) and Gibbs sampling. In this study, for understanding the dependency between the inputs, we utilized protein interaction information along with SGD and Gibbs sampling. A characteristic property of the genes is that their protein products have strong physical interactions with each other. Hence the protein interaction information is utilized for inferring the dependency between the inputs.

In this study, the generative model is used as an ensembling model that takes different weak supervision solutions as inputs and infers a probabilistic solution by considering their interrelated dependencies. Hence, the performance of the generative model depends upon the quality of the input solutions along with the generative model architecture. For weak supervision sources, we have utilized two types of solutions. Each type of solution has different role in generating the final solution. The advantages of different weak supervised solutions are described as follows.

1. MOO-based solutions: These weakly supervised solutions are generated after applying a MOO-based clustering algorithm on the gene expression datasets. The proposed MOO-based clustering technique generates the solutions after optimizing three objective functions (described in subsection ***MOO-based clustering***). In recent literature^39,46^, MOO-based clustering has been found to be a powerful technique in solving a wide variety of problems. The MOO-based clustering solutions are generated by programmatic rules and do not utilize any ground truth information. Hence, protein interaction information is being used as the weighting factor of the accuracy for each solution. The addition of the protein interaction information as the weighting factor with the generative model improves the performance of the model. The detailed comparative analysis of the experimental results (described in subsection ***Experiment Results***) proves the effectiveness of using protein interaction information in improving gene clustering.
2. GO-based solutions: Along with the MOO-based solutions, we generated three more weakly supervised solutions by utilizing Gene Ontology. These three solutions are generated by considering three biological aspects of the gene ontology namely molecular function (MF), biological process (BP) and cellular component (CC). These GO-based solutions are generated by accessing an external knowledge base. These solutions act as nearly ground truth, hence leveraging the performance of the model.

In this study, we have integrated these weak supervision solutions by using three variants of the generative model, namely ${{\mathbb{M}}}_{1}$, ${{\mathbb{M}}}_{2}$ and ${{\mathbb{M}}}_{3}$. Here, ${{\mathbb{M}}}_{1}$ refers to a vanilla model where only the MOO-based solutions are used to infer the final probabilistic model. The proposed MOO-based clustering technique generates a significant amount of optimized solutions. These optimized solutions guide us to infer the final probabilistic labels using the vanilla model ${{\mathbb{M}}}_{1}$. However, in model ${{\mathbb{M}}}_{1}$, it is assumed that all the MOO-based solutions have equal weights in regard to their accuracies which lead to misjudging the quality of the final inferred solutions. Hence, to assign the appropriate weights to different MOO-based solutions, we make use of protein-protein interaction information. In this regard, we develop ${{\mathbb{M}}}_{2}$ model where protein interaction information is processed for inferring the weight of each solution. In the above two models (${{\mathbb{M}}}_{1}$ and ${{\mathbb{M}}}_{2}$), we did not take into account any ground truth information about the genes for inferring the final probabilistic labels. To enhance the biological relevance of the final solution, along with the MOO-based solutions, we have added three GO-based solutions obtained from a human-curated database. This database refers to Gene Ontology Consortium (GOC) which is the world’s largest knowledge-base of gene functions. The three solutions are generated by performing an enrichment analysis on the GOC using the PANTHER (**P**rotein **AN**alysis **TH**rough **E**volutionary **R**elationships) classification system. In this regard, finally, we develop an integrated generative model (${{\mathbb{M}}}_{3}$) which exploits GO-based solutions along with the MOO-based solutions. As the GO-based solutions are generated by utilizing the human-curated databases, the integration of these solutions enhances the performance of the ${{\mathbb{M}}}_{3}$ model.

Keeping the above arguments in mind, an important question may arise as why we have not used GO-based solutions exclusively as they are considered as near ground truth. The reason behind this is as follows:

- The number of GO-based solutions that we can obtain is quite low. Hence inferring the final solution by considering only these solutions is prone to over-fitting.
- The PANTHER classification system does not classify all the genes as Gene Ontology Consortium may not contain the information for all the genes. Thus, the GO-based solutions do not provide labels for those unmapped genes.

For the above two reasons, we did not use only GO-based solutions for inferring the final solution. The integration of two types of solutions helps us in improving the overall performance of the generative model in terms of three quality measures, BHI, BSI metrics and Silhouette score. As the MOO-based solutions are reasonable in number and pro-grammatically validated, these solutions help us to capture the interrelation between the solutions. On the other hand, GO-based solutions help us to incorporate gene enrichment analysis information within the proposed generative model. In a nutshell, these two types of solutions are of equal importance in enhancing the model performance. To validate the performance of our proposed generative model in terms of biological relevance, we have done a biological analysis of the obtained gene clusters. Here we provide a thorough assessment of the acquired gene clusters ’ biological enrichment. by GOTERMMAPPER (https://go.princeton.edu/cgi-bin/GOTermMapper). This finding confirms that the genes of a cluster detected by the proposed gene clustering method are more engaged in the same biological mechanism/function compared to the genome’s remaining genes.

## Methods and Materials

For the proposed weakly supervised ensembling technique, the key steps are summarized as follows

- In the first step, we filtered out the redundant genes from the gene expression profiles. The remaining genes are used for the subsequent steps.
- The remaining genes are used to generate the ***base partitions***(BP) by exploiting two different approaches.
  1. In the first step, we acquired the solutions by using ***weak supervision technique*** effectively. In this respect, we used a clustering technique based on multi-objective optimization (MOO).
  2. In the second approach, we took ***Gene Ontology (GO)***^38^ into consideration for generating partitioning solutions.
- Finally, to obtain the ***consensus partitioning***(solution), we utilized a generative model considering the protein protein interaction information.

**Figure 1:**
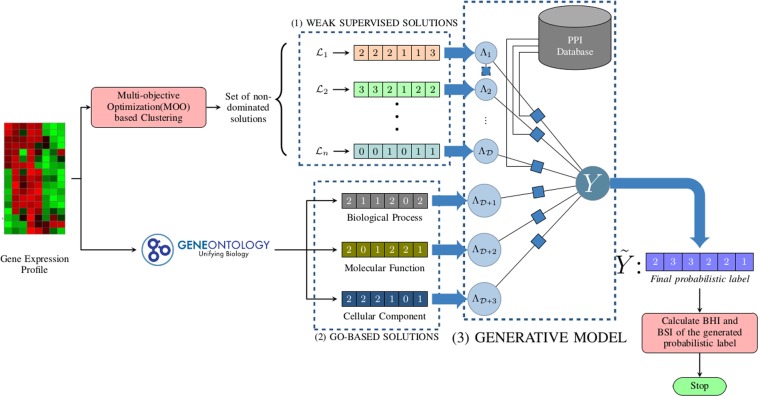
An overview of the proposed weak supervision based gene clustering architecture. (1) Solutions obtained from MOO-based clustering which considers as a weak supervision source. (2) Solutions obtained by exploiting Gene Ontology. (3) Protein interaction information is integrated with the generative model to generate the final probabilistic label. The integration of protein interaction information with the generative model is further pictorially described in Fig. 3.

Figure 1 represents the schematic flowchart of our proposed weakly supervised ensemble based gene clustering technique. The details of the above key steps are described in the subsequent subsections.

### Preprocessing of the dataset

In recent years, gene expression profiles (microarray) have become one of the backbones for the enhancement in the computational genomics. Though there are plenty of microarray datasets, the main bottleneck is to get the biological insights by analyzing those datasets. Let, a gene expression profile (*G*) be represented as a 2D-matrix where $G\in {{\mathbb{R}}}^{N\times F}$. Here, *N* represents the number of genes, {*g*_1_, *g*_2_, …, *g*_*N*_}, and each gene is represented as a *F*-dimensional feature (sample) vector. Among the *N* genes, not all genes are relevant under the pathogenic studies. The genes which are up- and down-regulated^47^ between different tissue samples are important for analyzing any disease. These down- and up-regulated genes are called differentially expressed (DE) genes^48^.

In this study, to filter out the differentially expressed (DE) genes, a statistical test is used. Firstly, we filtered out the genes based on the variances across the samples^48^. Finally, bootstrapped-*p* value^47^ is used as a threshold to filter out the statistically significant genes. In this work, the genes $(\widehat{G}\in \{{g}_{1},{g}_{2},\ldots ,{g}_{\widehat{N}}\})$ with bootstrapped-*p* values less than 0.05 are considered as statistically significant and used for further data analysis. We have applied this statistical preprocessing step on three real-life NCBI’s GEO datasets, namely B-CLL chronic lymphocytic leukemia^49^, Interstitial lung disease (ILD)^50^, and Prostate dataset^51^. Initially, B-CLL dataset (*N* = 12,625) contains 11 B-CLL stable samples and 10 clinically progressive disease-related samples. Similarly, ILD dataset (*N* = 54,675) has 29 samples (6 normal and 23 ILD-related) and prostrate dataset (*N* = 20,000) has 104 (70 disease-related and 34 normal) samples. After prepossessing all three datasets, the total number of differential genes of the datasets are 4,656 (${\widehat{N}}_{B-CLL}$), 18,144 (${\widehat{N}}_{ILD}$), and 2,424 (${\widehat{N}}_{prostrate}$). The prepossessed datasets are available in **supporting online repository**.

### Generation of weak supervised solutions

In any ensembling technique, generating diverse base partitionings is one of the crucial steps to generate an improved consensus partitioning. In this study, for creating base partitionings, we exploited *weak supervision* technique. In weak supervision, rather than consulting with trained *subject matter experts* (SME), the solutions (labels) are generated programmatically by analyzing heuristic patterns^52,53^, crowd-sourced data^54,55^ and external knowledge base^25,56^. Thus data generated by weakly supervised sources are cheaper, noisier and have less accuracy and coverage. Ideally, to increase accuracy and coverage, weak supervised solutions are combined to generate the final probabilistic solution.

In this study, to maintain the diversity of weak supervision labels, we have used two approaches, first one is a multi-objective optimization (MOO) based clustering technique, and another approach is to exploit Gene Ontology. In MOO-based technique, weak supervised solutions are generated programmatically by analyzing data patterns, whereas the external knowledge database is exploited to generate Gene Ontology-based solutions. In this article, *weak supervised solutions* are analogous to *weak supervised labels*. The detailed description of creation of these two types of weak supervised solutions is presented in the subsequent subsections.

#### MOO-based clustering

**Figure 2:**
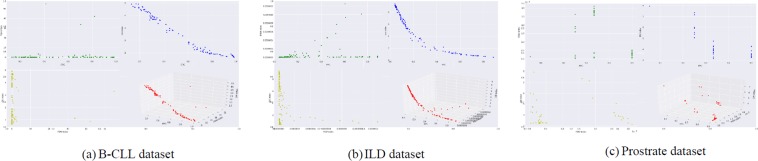
Pareto optimal fronts that contain the non-dominated solutions obtained from the multi-objective optimization technique. These non-dominated solutions are considered as the weak supervised solutions of the generative model.

In this step, weak supervision labels are generated by a proposed MOO-based clustering technique^20^ which determines a set of partitions by optimizing some cluster quality measures simultaneously. The search capability of a multiobjective based optimization strategy is utilized for the purpose of optimization. Let the available gene expression profiles be denoted by $(\widehat{G})$. Let, ${\mathbb{MO}}$ represent the proposed MOO-based clustering technique which takes input $\widehat{G}$ and generates a set of non-dominated solutions {*S*_1_, *S*_2_, …, *S*_*D*_} by simultaneously optimizing three objective functions {*f*_1_, *f*_2_, *f*_3_}. Hence, mathematically we can describe that

1 $${\mathbb{MO}}(\widehat{G})=\{{S}_{1},{S}_{2},\ldots ,{S}_{D}\}| O({f}_{1},{f}_{2},{f}_{3})$$

where function *O*, simultaneously optimizes all three objective functions. In the study, non-dominated sorting genetic algorithm II (NSGA-II)^57^ is used as the underlying multi-objective optimization technique and fuzzy c-means clustering^58^ is used to assign labels for different genes $\{{g}_{1},{g}_{2},\ldots ,{g}_{\widehat{N}}\}$. In order to exploit the search space extensively, variable length chromosomes are used along with three genetic operators. These three genetic operators are crossover, mutation and selection. After applying these three genetic operators, new population is generated. In each generation, we simultaneously optimize three objective functions and the best solutions are selected after application of non-dominated sorting and crowding distance operators. These three objective functions are: *f*_1_ := Fuzzy Partition Coefficient (FPC)^58^, *f*_2_ = Pakhira-Bandyopadhyay-Maulik index (PBM index)^59^ and *f*_3_ = DB index^60^ and finally a set of non-dominated solutions {*S*_1_, *S*_2_, …, *S*_*D*_} are generated. These non-dominated solutions are placed in the Pareto optimal front which is shown in Fig. 2.

For each non-dominated solution, a label is generated (*L*_*i*_) by a corresponding labeling function (*λ*_*i*_), i.e., ${\lambda }_{i}(\widehat{G})={L}_{i}\ | \ i\ \in [1,D]$. These labels, {*L*_1_, *L*_2_, …, *L*_*D*_} are generated in a programmatic manner and considered as the weak supervised labels. These weak supervised labels are then encoded into the proposed generative model. The set of non-dominated solutions, which created the Pareto optimal front is shown in Fig. 2.

#### GO-based solutions

To maintain diversity among the weak supervision sources, along with the MOO-based solutions, we exploited Gene Ontology (GO) for generating the weak supervision sources. Gene Ontology (GO)^38^ is the world’s largest ***knowledge base*** that contains the information about gene functionality. This knowledge base is both human-readable and machine-readable and is a foundation for computational analysis of large-scale molecular biology and genetic experiments in biomedical research. In this study, this functional knowledge base of genes is considered as a weak supervision source. To generate the weak supervised solutions, Gene Ontology performs enrichment analysis on the preprocessed gene expression profile ($\widehat{G}$). The enrichment results reveal the associations between gene sets and GO terms. The enrichment analysis is carried out by PANTHER(**P**rotein **AN**alysis **TH**rough **E**volutionary **R**elationships)^61^ classification system. PANTHER classification is a result of subject matter expert’s (SME) annotation/curation.

In this task, PANTHER generates gene labels with respect to three biological aspects, namely, molecular function (MF), biological process (BP) and cellular component (CC). Here, these three aspects are considered as the three weak supervised labeling functions, i.e., *λ*_*MF*_, *λ*_*BP*_, *λ*_*CC*_. In each weak supervised labeling function, a list of shared GO terms (*GO*_1_, *GO*_2_, …, *GO*_*L*_) are generated where each shared GO term consists of a set of genes, i.e., ${GO}_{i}\ =\ \left\{\left({g}_{1}^{i},{g}_{2}^{i},\ldots ,{g}_{P}^{i}\right)| i\in [1,L]\right\}$ where *P* represents the number of genes of a particular shared GO term. Each labeling function generates multi-label solutions (*L*_*MF*_, *L*_*BP*_, *L*_*CC*_) where the genes associated with particular GO term are assigned a unique label.

These solutions (*L*_*MF*_, *L*_*BP*_, *L*_*CC*_) are also considered as weak supervised solutions along with MOO-based solutions (described in the previous subsection) and are considered for constructing the consensus partitioning using the proposed generative model. In these solutions, the genes are labelled according to their shared GO term (*GO*_*i*_∣*i* ∈ [1, *L*]) based classification. Since not all genes are mapped in the Gene Ontology Consortium, we have considered that *λ*_*MF*_, *λ*_*BP*_, *λ*_*CC*_ are abstaining from labelling those genes. Hence, in each of these GO-based solutions (*L*_*MF*_, *L*_*BP*_, *L*_*CC*_), some genes are kept unlabelled. Though some of the genes are unlabelled in these solutions, the labels of remaining genes can be considered near to ground truth. As these GO-based solutions are generated by exploiting biomedical knowledge base, these solutions help in increasing the performance of the generative model. Also in the result section, we have shown that the addition of these GO-based solutions improves the performance of the generative model compared to traditional generative model.

### Inception of generative model

The core concept of the proposed architecture is the generative model. The developed generative model takes different weak supervision sources and finally infers a list that contains probabilistic labels for all the samples. The key challenge of the approach is in determining how to integrate weak supervision labels which have unknown correlations, accuracies and different levels of granularity. Hence, this integration phase acts as a critical step in shaping performance of the model. In this regard, the generative model plays an essential role in overcoming this roadblock. The performance of such a generative model is highly dependent on its structure, as the proper structure helps in inferring the accurate correlations between weak supervision labels.

In this study, we developed a generative model which acts as a framework for integrating weak supervision sources to infer labels of the genes. To accomplish this, we modified a popular generative model named Snorkel^28^ by utilizing protein protein interaction information. The workflow of Snorkel is different from traditional approaches and is built upon a new machine learning paradigm called data programming^62^. Snorkel offers a trade-off between training time and performance of the model. Also, the structure of Snorkel helps in predicting accurate class labels automatically. The application of Snorkel in top industries, research labs and government agencies show its wide-ranging capabilities in building improved models.

Motivated by the success of Snorkel in a wide range of domains, we utilized a modified version of it for improving gene clustering. In our case, we have modified the *generative model* part of Snorkel. Let, the generative model *p*_*θ*_ integrate the weak supervision labeling function obtained from MOO-based clustering and Gene Ontology, i.e., *λ*_1_, *λ*_2_, …, *λ*_*D*_, *λ*_*MF*_, *λ*_*BP*_, *λ*_*CC*_. In general, the labelling function of the generative model are autonomous or uncorrelated to each other. But in the proposed generative model, we considered the statistical dependencies between the labelling functions. This dependence enhances the generative model’s predictive accuracy. Finally, each of the data points (gene) is generated as a latent variable by the generative mathematical model.

The proposed generative model (*p*_*θ*_) designed as a factored graph (${\mathbb{G}}$)^63^ which is a sort of probabilistic graphic model that includes two kinds of nodes. These two kinds of nodes are ***evidence variable*** and ***factors***. The ***factors*** describe the relationships in the factor graph between the ***estimate variables***.

In this work, the *D* labels {*L*_1_, *L*_2_, …, *L*_*D*_} acquired from MOO-based clustering and three Gene Ontology-based labels {*L*_*MF*_, *L*_*BP*_ and *L*_*CC*_} are interpreted as the ***evidence variables*** of factor graph ${\mathbb{G}}$. These *D* + 3 labels helps to generate a label matrix $\Lambda \in {\{0,1,\ldots ,C\}}^{\widehat{N}\times (D+3)}$ which is further fed to the probabilistic generative model, *p*_*θ*_. This probabilistic model predicts probabilistic labels, $Y=\left\{\widetilde{{y}_{1}},\widetilde{{y}_{2}},\ldots ,{y}_{\widehat{N}}\right\}$, using three kinds of ***factors***. The proposed generative model is represented as *p*_*θ*_(Λ, *Y*) and the three ***factors*** are defined as

- ***Labeling propensity*** : $${\phi }_{i,j}^{Lab}(\Lambda ,Y)=1\{{\Lambda }_{i,j}\ne \varnothing \}$$
- ***Accuracy*** : = $${\phi }_{i,j}^{Acc}(\Lambda ,Y)=1\{{\Lambda }_{i,j}\ne {y}_{i}\}$$
- ***Pairwise correlations*** : = $${\phi }_{i,j,k}^{Corr}(\Lambda ,Y)=1\{{\Lambda }_{i,j}={\Lambda }_{i,k}\}$$

where Λ_*i*,*j*_ represent the element of the label matrix, Λ, and is defined as Λ_*i*,*j*_ = *λ*_*j*_(*g*_*i*_). We calculated these three factors for a particular gene, *g*_*i*_, and concatenated into a vector *ϕi*(Λ, *Y*) for all *D* + 3 labeling functions. The proposed probabilistic generative model is described as

2 $$\begin{array}{lll}{p}_{\theta }(\Lambda ,Y) & = & {\xi }^{-1}exp\left({\sum }_{i=1}^{\widehat{N}}{\theta }^{T}{\phi }_{i}(\Lambda ,{y}_{i})\right)\\  & = & {\xi }^{-1}exp\left({\sum }_{i=1}^{\widehat{N}}{\theta }^{T}{\sum }_{j=1}^{D+3}\left({\phi }_{j}^{Acc}(\Lambda ,{y}_{i})+{\phi }_{j}^{Lab}(\Lambda ,{y}_{i})+{\phi }_{j}^{Corr}(\Lambda ,{y}_{i})\right)\right)\\  & = & {\xi }^{-1}exp\left({\sum }_{i=1}^{\widehat{N}}{\sum }_{j=1}^{D+3}\left({\theta }^{T}{\phi }_{j}^{Acc}(\Lambda ,{y}_{i})+{\theta }^{T}{\phi }_{j}^{Lab}(\Lambda ,{y}_{i})+{\theta }^{T}{\phi }_{j}^{Corr}(\Lambda ,{y}_{i})\right)\right)\end{array}$$

In the above Eq. 2, *ξ* is the normalizing constant. In case of a conditionally independent model, we estimate the parameter, *θ*, by minimizing the negative log marginal likelihood for the observed label matrix, Λ

3 $$\mathop{{\rm{\arg }}\,{\rm{\min }}}\limits_{\theta }-\,\log \,\sum _{Y}{p}_{\theta }(\Lambda ,Y)$$

In a general generative model, the values of the parameters (*θ*) are estimated by Eq. 3. These parameters estimate the strength of the three factors of the generative model. Among the three factors, the parameters for two factors (*ϕ*^*Lab*^, *ϕ*^*Corr*^) are estimated by Eq. 3 and for the remaining factor (*ϕ*^*Acc*^), the parameters are calculated by utilizing protein protein interaction information. In this study, the accuracy parameter values for the MOO-based solutions are generated by utilizing protein protein interaction information, and the accuracy parameter values for GO-based solutions are generated by Eq. 3. The accuracy parameter for a particular non-dominated solution (*S*_*i*_) is represented as ${\theta }_{i}^{PPI}$. Hence the Eq. 2 can be written as

4 $${p}_{\theta }(\Lambda ,Y)={p}_{\theta }^{MOO}(\Lambda ,Y)+{p}_{\theta }^{GO}(\Lambda ,Y)$$

where ${p}_{\theta }^{MOO}(\Lambda ,Y)$ and ${p}_{\theta }^{GO}(\Lambda ,Y)$ are described as

5 $${p}_{\theta }^{MOO}(\Lambda ,Y)={\xi }^{-1}exp\left({\sum }_{i=1}^{\widehat{N}}{\sum }_{j=1}^{D}\left({\theta }_{j}^{PPI}{\phi }_{j}^{Acc}(\Lambda ,{y}_{i})+{\theta }^{T}{\phi }_{j}^{Lab}(\Lambda ,{y}_{i})+{\theta }^{T}{\phi }_{j}^{Corr}(\Lambda ,{y}_{i})\right)\right)$$

6 $${p}_{\theta }^{GO}(\Lambda ,Y)={\xi }^{-1}exp\left({\sum }_{i=1}^{\widehat{N}}{\sum }_{j=1}^{3}\left({\theta }^{T}{\phi }_{D+j}^{Acc}(\Lambda ,{y}_{i})+{\theta }^{T}{\phi }_{D+j}^{Lab}(\Lambda ,{y}_{i})+{\theta }^{T}{\phi }_{D+j}^{Corr}(\Lambda ,{y}_{i})\right)\right)$$

**Figure 3:**
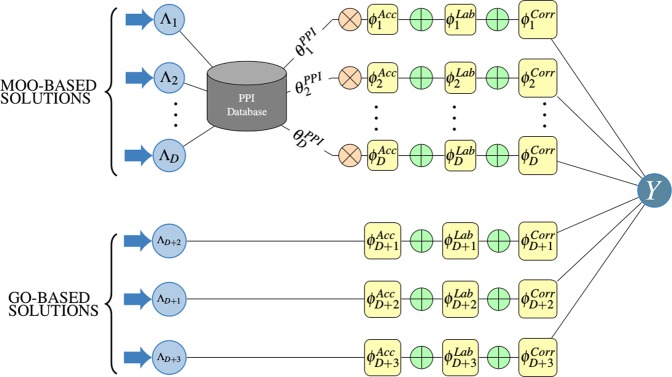
The underlying factor graph of the proposed generative model.

The integration of protein interaction with generative model along with underlying architecture is shown in Fig. 3. The parameter ${\theta }_{i}^{PPI}$ is generated by exploiting an updated protein-protein interaction resource named **HitPredict**^64^. HitPredict is a resource of experimentally determined protein-protein interactions with reliability scores (*α*_*ij*_). This confidence score (*α*_*ij*_) of proteins *g*_*i*_ and *g*_*j*_ denotes the reliability of the interaction and is the geometric mean of annotation-based score and method based score. The annotation score is calculated based on the GO annotations of the interacting proteins. In the method score, score is calculated by considering the experimental evidence of the interactions between proteins. As *α*_*ij*_ takes into account both experimental support for the interaction and the genomic features of the interacting proteins, it is considered as a reliable source for exploiting the protein protein interactions.

For a particular non-dominated solution (*S*_*i*_) which consists of a set of clusters {*C*_1_, *C*_2_, …, *C*_*K*_}, ${\theta }_{i}^{PPI}$ is calculated by

7 $${\theta }_{i| i\in [1,D]}^{PPI}=\left\{\frac{1}{K}{\sum }_{r=1}^{K}CS({C}_{r})\hspace{2.84544pt}\left|\right.\hspace{2.84544pt}{C}_{r}\in {S}_{i};K=| {S}_{i}| \right\}$$

where for each ${C}_{r}^{th}$ cluster, *CS*(*C*_*r*_) is calculated as follows

8 $$CS({C}_{r})=\frac{1}{Q}\sum _{\begin{array}{c}(i,j)| \{{g}_{i},{g}_{j}\}\in {C}_{r}\\ ({g}_{i}\ne {g}_{j})\end{array}}{\alpha }_{i,j}\hspace{4.26773pt}\,where\,\hspace{4.26773pt}1\le Q{\le }^{{n}_{r}}{C}_{2}$$

where *n*_*r*_ represents the number of genes present in the cluster *C*_*r*_; *Q* represents the number of protein protein interactions extracted from **HitPredict**^64^ for all the genes of ${C}_{r}^{th}$ cluster. As ${\theta }_{i}^{PPI}$ of a non-dominated solution (*S*_*i*_) is generated by utilizing the protein interaction information, ${\theta }_{i}^{PPI}$ helps to understand the biological significance of the solution. This PPI information replaces the default weighting factor for each labelling function in order to improve the accuracy of results obtained from the generative model.

## Scalability of the Proposed Approach

The proposed approach consists of two subtasks (*generating the weak supervised solutions* and *inferring labels from those generated solutions*) that correctly infer the probabilistic labels of the genes. In this section, we discuss about the time complexities of different subtasks and along with overall time complexity of the proposed approach.

- For generating the weak supervised solutions, we use our proposed multi-objective optimization based clustering technique. NSGA-II is used as the underlying multi-objective optimization technique which has a time complexity of *O*(*mn*^2^). Here *n* is the size of the population, and *m* is the number of objective functions. Here *m* equals to 3, and the complexities of computing different objective functions are as follows: *Fuzzy Partition Coefficient(FPC) index :O*(*n*) *Pakhira-Bandyopadhyay-Maulik index(PBM index) :O*(*n*) *DB index :O*(*n*) Therefore the overall time complexity of the algorithm is
  9 $$\begin{array}{lll}{T}_{1}(n) & \le  & [{C}_{1}(n)+{C}_{2}(n)+{C}_{3}(n)]+{C}_{4}({n}^{2})\\  & \le  & {C}_{5}(n)+{C}_{4}({n}^{2})\\  & \le  & {C}_{4}({n}^{2}\ log(n))\\ {T}_{1}(n) & = & O({n}^{2})\end{array}$$
- For inferring the probabilistic label, we modified a popular generative model named Snorkel^28^. The time complexity of snorkel is *T*_2_(*n*) = *O*(*n*log*n*)^65^

Hence, the overall time complexity of the proposed approach is

10 $$\begin{array}{lll}T(n) & = & {T}_{1}(n)+{T}_{2}(n)\\  & \le  & {C}_{6}({n}^{2})+{C}_{7}(n\,log\,n)\\  & \le  & {C}_{8}({n}^{2})\\ T(n) & = & O({n}^{2})\end{array}$$

Hence, the proposed approach runs in polynomial time. From this time complexity analysis, we can infer that the proposed approach is robust irrespective of the size of the dataset. In the current paper, the proposed technique are applied on the datasets with varied number of genes(range from 2000 to 18000) and samples(range from 21 to 104). Results also prove that the proposed system is robust irrespective of the dataset size.

## Conclusion

In this paper, we properly utilize different weak supervision sources using a newly developed generative model for improving gene clustering. In this work, rather than using any labelled data, we utilize different weak supervised sources to perform the desired task. Hence, our model overcomes the bottlenecks related to subject matter experts and manual annotation time. The proposed generative model utilizes weak supervision sources along with protein interaction information for inferring the correlations and dependencies of different sources. In this study, for weakly supervised sources, we utilized a multi-objective optimization-based clustering technique along with three gene ontology-based three solutions. These GO-based solutions help to improve the performance of the generative model as these are generated by utilizing the biomedical knowledge base. Also, the use of protein interaction information as the latent variable of the proposed generative model helps to leverage the performance of the proposed model. The obtained results prove the superiority of the proposed method than other existing methods in terms of biological homogeneity index (BHI), biological stability index (BSI) and Silhouette index. Finally, biological analyses are conducted to validate the obtained results.

In the future, we will use the proposed ensemble method to perform various biomedical functions where the real class labels are not available. We will also attempt to develop an enhanced version of the ensemble method by modifying the generative model’s variables that will be able to perform the job more correctly.

## Data Availability

The source code and all datasets used in this study are available at https://github.com/sduttap16/PPI_Generative.
